# Supplementary material for: Pairing a high-resolution statistical potential with a nucleobase-centric sampling algorithm for improving RNA model refinement
Source: Nat Commun. 2021 May 13;12:2777. doi: 10.1038/s41467-021-23100-4 (PMC8119458; doi:10.1038/s41467-021-23100-4)
Supplement: Supplementary file 1 — Supplementary Information [file 41467_2021_23100_MOESM1_ESM.pdf]

Supplementary information

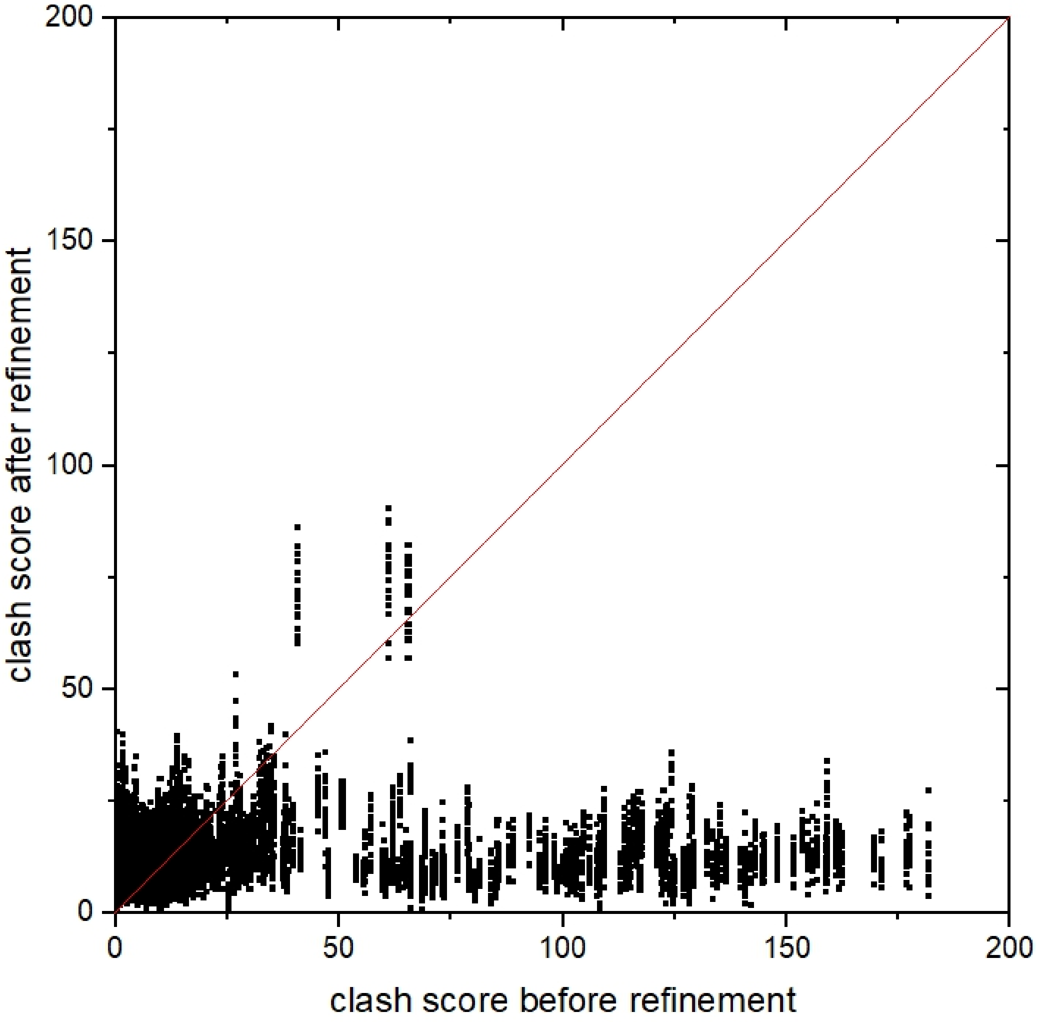

Supplementary Figure S1. The clash scores from MolProbity before (X-axis) and after (Y-axis) refinement for all RNA puzzle structures. The red solid line indicates the line with unchanged clash scores after refinement.

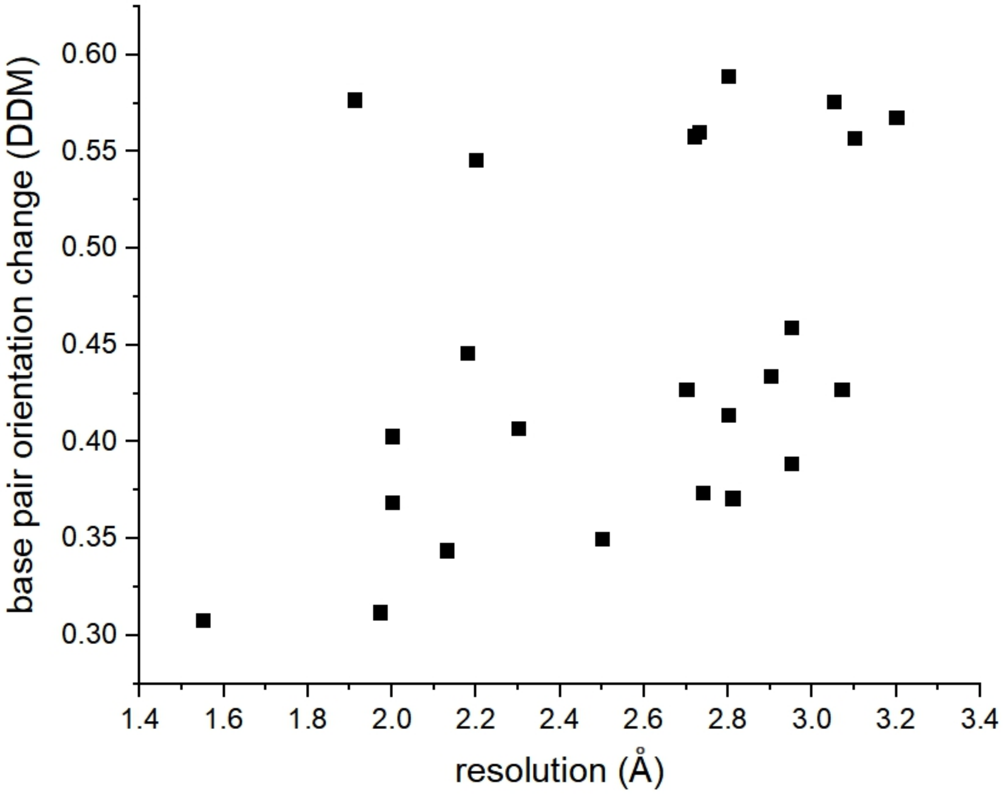

Supplementary Figure S2, the change of base pair orientation measured by DDM after BRiQ refinement of the native structures of RNA puzzle targets as a function of their X-Ray structure resolutions.

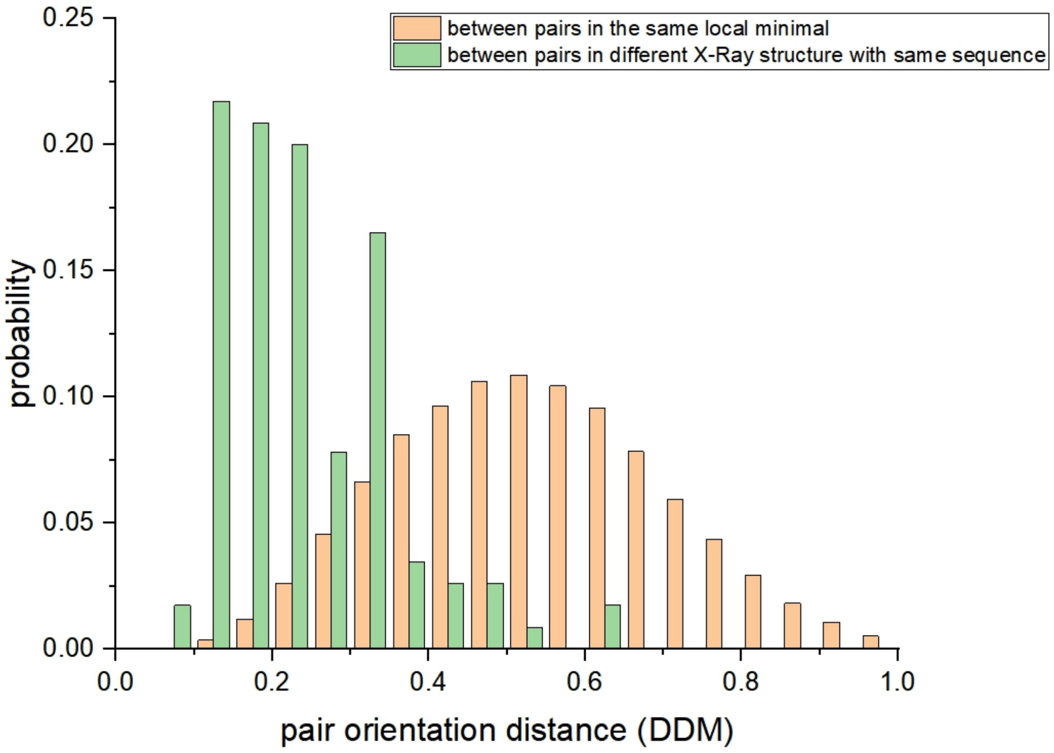

Supplementary Figure S3, the contribution to the base-pair probability from different structures with the same sequences and other structures in the same local minimum as a function of the distance between two pairs according to DDM.

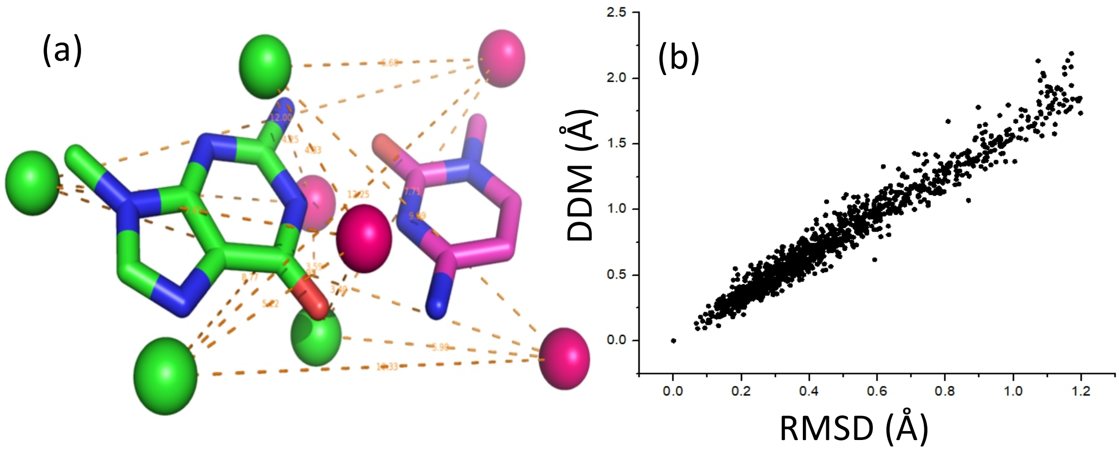

Supplementary Figure S4. a) Four points in the local base coordination system are employed to measure the relative orientation between two bases. b) DDM, the mean squared distance between the four points of two separate bases has a high correlation coefficient with the RMSD after optimizing the locations of four points.

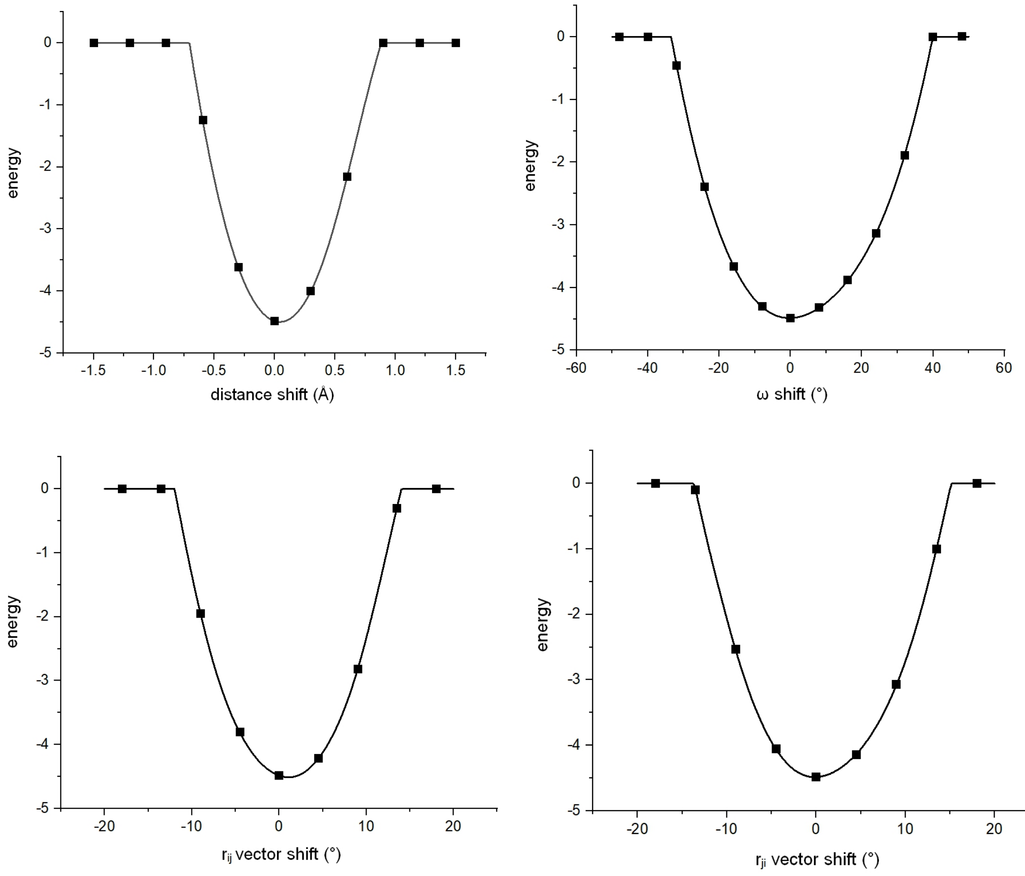

Supplementary Figure S5: An example of the statistical energy score for the AU pair with a single degree of freedom variation around the local minimum (distance, w, $\mathbf{r}_{ij}$ and $\mathbf{r}_{ji}$ vector angle shifts). The black squares are the grid points. The energy values on grid points were pre-calculated and stored in an energy table. The final energy value of a target base pair was calculated by the interpolation from nearest 36 grid points (nearest two points in distance and dihedral angle dimensions, nearest three points in $\boldsymbol{r}_{ij}$ and $\boldsymbol{r}_{ji}$ dimension, 2*2*3*3=36).

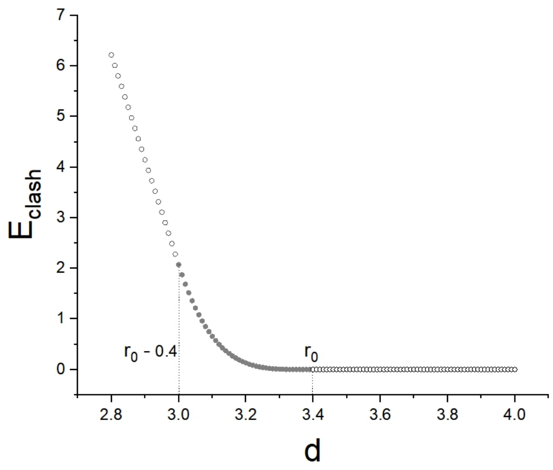

Supplementary Figure S6. $E_{clash}(d)$ as a function of the atomic distance. Here $k_{clash} = 3$.

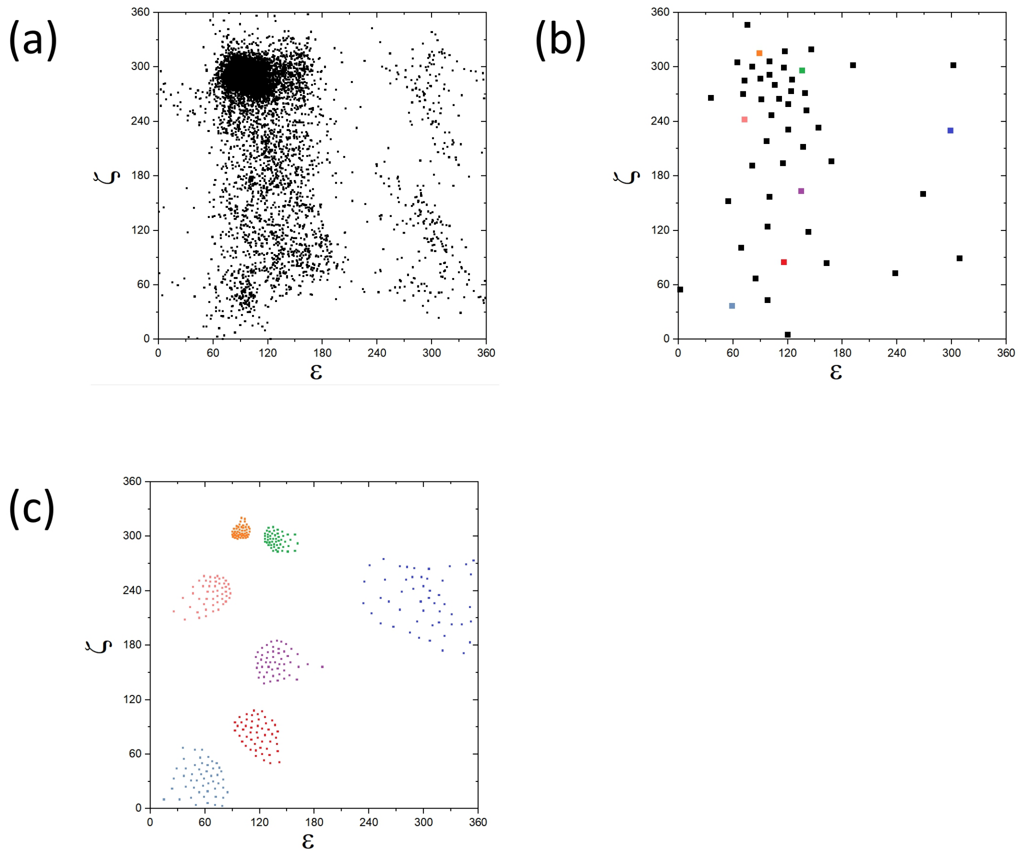

Supplementary Figure S7 (a) The conformational distribution in the ε-ζ torsional space in the database (b) the 50 representative points (c) 50 sub-representative points for several selected representative points.
