# Supplementary material for: Pairing a high-resolution statistical potential with a nucleobase-centric sampling algorithm for improving RNA model refinement
Source: Nat Commun. 2021 May 13;12:2777. doi: 10.1038/s41467-021-23100-4 (PMC8119458; doi:10.1038/s41467-021-23100-4)
Supplement: Supplementary file 3 — Description of Additional Supplementary Files [file 41467_2021_23100_MOESM3_ESM.pdf]

Description of Additional Supplementary Files

File Name: Supplementary Data 1
Description: The test set of 48 RNA motifs with lower case letters indicating fixed regions predefined in Rosetta-SWM.

File Name: Supplementary Data 2
Description: The RNA puzzle test set along with RMSD and DI values for the best pre-refinement model, the best model in BRiQ-predicted models and the best model in BRiQ-sampled models for each RNA puzzle experiment.

File Name: Supplementary Data 3
Description: The change of DDM values before and after BRiQ refinement for Watson-Crick, non-Watson-Crick, and stacking base pairs.

File Name: Supplementary Data 4
Description: Refinement of 2000 FARFAR2-predicted models for 12 RNA Puzzles by BRiQ. Performance is compared by using the best model (smallest RMSD and DI) within top 1% and top 5% predicted by FARFAR2 and BRiQ energy scores.
